# Supplementary material for: Multidimensional T1 − T2 Relaxation Imaging In vivo on a Portable 0.064 T MRI Scanner
Source: bioRxiv. 2026 Sep 9:2026.09.04.749459. Preprint. [Version 1] doi: 10.64898/2026.09.04.749459 (PMC13622502; doi:10.64898/2026.09.04.749459)
Supplement: Supplementary file 1 [file media-1.pdf]

Supporting Information

Multidimensional T_1-T_2 Relaxation Imaging *In Vivo* on a Portable 0.064 T MRI Scanner

Ella Wilczynski, Kulam Najmudeen Magdoom, Alexandru V. Avram, Nathan H. Williamson, Dan Benjamini, Silvina G. Horovitz, and Peter J. Basser

Table S1

Quantitative MRI phantom relaxation data summary for individual T_1 and T_2 fitting and joint T_1-T_2 fitting. MRI reference values are from previously reported 0.064 T measurements [1], and NMR reference values are shown where available [2].

			**Mono-exponential**				**Joint distribution**				**MRI reference**		**NMR reference**			
**Tube**	**Content**	**Conc.**	**T_1 (ms)**		**T_2 (ms)**		**T_1 (ms)**		**T_2 (ms)**		**T_1 (ms)**	**T_2 (ms)**	**T_1 (ms)**		**T_2 (ms)**	
		(mM)	Mean	SE	Mean	SE	Mean	SE	Mean	SE	Mean	Mean	Mean	SE	Mean	SE
7	$MnCl_2$	0.135	352.66	6.16	173.92	1.06	344.01	5.33	176.00	0.67	336	180	343.27	0.53	185.37	2.04
8	$MnCl_2$	0.193	266.63	3.15	128.86	0.81	252.29	4.35	128.85	0.64	244	131	—	—	—	—
9	$MnCl_2$	0.277	177.03	0.92	94.42	1.22	175.64	0.97	89.95	0.79	165	95	168.03	0.65	88.98	0.29
10	$MnCl_2$	0.428	112.71	0.43	63.51	1.11	115.52	0.44	60.93	0.96	108	64	—	—	—	—
11	$MnCl_2$	0.556	84.26	0.56	52.87	0.87	87.80	0.88	46.41	0.87	82	50	78.74	0.40	44.18	0.32
12	$MnCl_2$	0.790	56.34	0.77	36.45	1.34	62.97	0.92	34.93	0.62	56	35	—	—	—	—
2	PVP (O)	50%	302.22	6.13	259.34	5.98	302.26	6.10	289.63	6.14	290	270	—	—	—	—
10	PVP (I)	50%	328.35	4.26	276.09	3.56	343.81	3.31	311.03	3.93	300	290	—	—	—	—
8	$NiCl_2$	7.74	191.44	0.77	129.01	1.27	194.84	0.40	129.76	1.05	200	187	—	—	—	—
9	$NiCl_2$	11.3	131.94	0.54	126.99	0.76	133.38	0.51	122.47	0.70	141	141	138.70	0.29	137.60	0.36
10	$NiCl_2$	16.5	86.66	0.55	98.69	1.03	89.14	0.80	94.23	0.83	97	100	—	—	—	—
11	$NiCl_2$	23.3	60.43	0.62	75.74	1.21	63.65	0.99	68.32	0.94	69	76	69.79	0.22	70.98	0.24
12	$NiCl_2$	32.7	41.39	0.42	54.70	0.80	43.01	0.57	48.02	0.47	49	55	—	—	—	—

Notes: Concentrations are reported in mM except for PVP, which is reported as a percentage. O = outer; I = inner. MRI and NMR columns contain reference relaxation values from the indicated sources. "—" indicates that no reference value was available.

References

1. Ljungberg E, Padormo F, Poorman M, et al. Characterization of portable ultra-low field MRI scanners for multicenter structural neuroimaging. *Human Brain Mapping.* 2025;46:e70217. doi:10.1002/hbm.70217.

2. Martin MN, Jordanova KV, Kos AB, Russek SE, Keenan KE, Stupic KF. Relaxation measurements of an MRI system phantom at low magnetic field strengths. *Magnetic Resonance Materials in Physics, Biology and Medicine.* 2023;36:477-485. doi:10.1007/s10334-023-01086-y.
